# Exploring the Clinical and Hematological Characteristics of Beta-Thalassemia Trait: A Comprehensive Analysis in a Tertiary Care Hospital Setting

**DOI:** 10.7759/cureus.61093

**Published:** 2024-05-26

**Authors:** Yogalakshmi E, Sudha Vasudevan, Sulochana Sonti, Kavitha Kannan, Chitra Srinivasan

**Affiliations:** 1 Pathology, Saveetha Medical College and Hospital, Saveetha Institute of Medical and Technical Sciences, Saveetha University, Chennai, IND

**Keywords:** anemia, rbc indices, mentzer index, hplc, beta thalassemia trait

## Abstract

Beta-thalassemia is one of the most common inherited hematological diseases caused by more than 350 mutations in the β-globin gene (HBB). Beta-thalassemia carrier or trait is associated with defects in one allele of the HBB gene. The majority of beta-thalassemia trait cases remain concealed in society and remain unnoticed as they are mostly asymptomatic or present with mild symptoms of anemia. There is a 25% chance of having children with beta-thalassemia major and a 50% chance of having carrier babies when two people with beta-thalassemia trait are married. Hence, it is important to identify the individuals with beta-thalassemia trait and provide counseling to understand the risks of pregnancy and its outcome.

Aim

To study the identification of beta-thalassemia trait cases along with their clinical findings and hematological correlation.

Materials and methods

Study Design

This was a retrospective study conducted at Saveetha Medical College and Hospital for a period of four years from January 2020 to December 2023.

Inclusion Criteria

Age group more than 18 years, antenatal mother, cases of anemia who were refractory to iron treatment, and screening of family members in the positive cases of beta-thalassemia trait.

Exclusion Criteria

History of blood transfusion within three months was excluded.

*Data Collection*

A total number of 837 cases were screened to rule out the presence of beta-thalassemia trait/hemoglobin (Hb) variants. A 2 mL of intravenous blood samples were collected in an ethylene diamine tetraacetic acid (EDTA) vacutainer tube and processed in a Sysmex XN 1000 (Hyogo, Japan: Sysmex Corporation) automated hematology analyzer. The hematological parameters were analyzed.

Statistical Analysis

The study included both descriptive and analytical characteristics. Mean and standard deviation (SD) were calculated for all the hematological parameters. Beta-thalassemia trait was diagnosed with an HbA2 level of more than 4.0% through high-performance liquid chromatography (HPLC) analysis.

Results

Among the 837 samples studied for HPLC screening, 74 (8.8%) cases were found to have beta-thalassemia trait. The age group included was from 18 years to 56 years. Of 74 cases studied, 32(43%) were females and 42(57%) were males. Among the 74 cases studied, the Mentzer index <13 was seen in 58 (78%) cases and the Mentzer index >13 was seen in 16 cases (22%). Thirty-four cases (46%) of beta-thalassemia traits presented to the hospital with a history of fever for evaluation and antenatal screening accounted for 19 cases (26%). The mean red blood cell (RBC) count was 5.5 million/cu.mm; mean corpuscular volume (MCV) was 63.8 fL; mean corpuscular hemoglobin (MCH) was 19.6 pg; red cell distribution width coefficient of variation (RDW-CV) was 17.8%. Among the 74 cases studied, 37(46%) cases had an Hb of more than 11 g/dL, 22 cases had mild anemia, 12 cases had moderate anemia, and three cases had severe anemia.

Conclusion

This study concluded that regular monitoring of the Mentzer index along with HPLC analysis is an effective approach in identifying beta-thalassemia trait cases and further providing genetic counseling among the couples that will help in reducing high-risk pregnancy and the birth of a child with thalassemia major.

## Introduction

Beta-thalassemia is one of the most prevalent inherited hematological diseases with about 350 mutations in the β-globin gene (HBB) located on chromosome 11 [1,2]. The hallmark of beta-thalassemia is either minimal or absence of β-globin chains in the hemoglobin (Hb) produced [3]. Beta-thalassemia has a global distribution as a consequence of population migration, and its prevalence fluctuates among different populations. It is more prevalent in the Mediterranean region, the Middle East, parts of North and sub-Saharan Africa, the Indian subcontinent, and Southeast Asia [4-6]. The overall prevalence of beta-thalassemia in India with an average of 3%-4% with an estimate that around 10,000-12,000 children are born every year with beta-thalassemia major and around 42 million are beta-thalassemia carriers [7]. The prevalence of the beta-thalassemia trait has variously been reported in Central India, which ranged between 1.4% and 3.4%, and in South India, the beta-thalassemia trait prevalence ranged between 8.5% and 37.9%. The frequency of beta-thalassemia major was reported to be between 2.3% and 7.47% [8]. Beta-thalassemia carrier or trait is associated with defects in one allele of the HBB gene. Mutations in the HBB gene lead to a defect in globin production [9].

Beta-thalassemia trait is usually asymptomatic or mildly shows anemia. When individuals with beta-thalassemia carriers marry another with the same, there is a 25% risk at each pregnancy of having children with beta-thalassemia major and a 50% risk of having carrier babies [10]. These thalassemia major children suffer from severe anemia, jaundice, stunted growth, and hepatosplenomegaly. These children need frequent blood transfusions, iron chelators to treat their iron overload, and bone marrow transplants. Beta-thalassemia major causes shorter life duration, end organ damage, and chronic anemia if left untreated. Reduction of the birth rate of beta-thalassemia major children can be achieved by adopting carrier screening for the population of high-risk, premarital individuals, antenatal mothers, and extended or immediate family members, providing genetic counseling [11]. The study aimed to identify beta-thalassemia trait cases along with its clinical findings and hematological correlation through various methods of analysis of hematological parameters such as Hb, red blood cell (RBC) indices, Mentzer index, and red cell distribution width (RDW), high-performance liquid chromatography (HPLC) screening for HbA2/F levels [12].

## Materials and methods

Study design

This was a retrospective study conducted in the Department of Pathology in our institute, a tertiary care center on the outskirts of Chennai, Tamilnadu, India. The study got approval from the ethical committee with an IEC Reference number 008/12/2022/ IEC/SMCH. The period of study included four years from January 2020 to December 2023.

Data collection

A total number of 837 cases were studied to rule out the presence of beta-thalassemia trait/Hb variants. A 2 mL of intravenous blood were drawn under sterile conditions, placed in a K2 ethylene diamine tetraacetic acid (EDTA) vacutainer, and processed in a Sysmex XN 1000 (Hyogo, Japan: Sysmex Corporation) automated hematology analyzer. The hematological parameters such as Hb, RBC count, mean corpuscular volume (MCV), mean corpuscular hemoglobin (MCH), mean corpuscular hemoglobin concentration (MCHC), RDW, total white blood cell count (TWBC), differential count (DC), and platelet count were analyzed. The Mentzer index was calculated by using the formula MCV/RBC count. HPLC was done by using a D-10 analyzer to determine the HbA2 and HbF percentages. Beta-thalassemia trait was diagnosed with an HbA2 level of more than 4.0%. The retention time of HbA2 levels was studied.

Inclusion criteria

Age group more than 18 years, antenatal mother, cases of anemia who were refractory to iron treatment, and screening of family members in the positive cases of beta-thalassemia trait.

Exclusion criteria 

History of blood transfusion within three months was excluded.

Statistical analysis 

This study included both descriptive and analytical characteristics. Age, gender, clinical findings, and demographic distribution were studied. Mean and standard deviation (SD) were calculated for all the hematological parameters.

## Results

Among the 837 samples studied for HPLC screening, 74 (8.8%) cases were found to have beta-thalassemia trait. The age group included was from 18 to 56 years and the mean age was found to be 27 years. The gender distribution of the beta-thalassemia trait is illustrated in Table 1.

**Table 1:** Gender distribution of beta-thalassemia trait cases

Number of cases positive for beta-thalassemia trait	Number of males positive for beta-thalassemia trait	Number of females positive for beta-thalassemia trait
74 (8.8%)	42 (57%)	32 (43%)

Among the 74 cases studied, the Mentzer index <13 was seen in 58 (78%) cases, and the Mentzer index >13 was seen in 16 cases (22%). The majority of beta-thalassemia traits presented to the hospital with a history of fever for evaluation were 34 cases (46%) and antenatal screening accounted for 19 cases (26%). The additional clinical manifestation of subjects with beta-thalassemia trait has been displayed in Table 2.

**Table 2:** Various clinical manifestations of beta-thalassemia trait cases

Clinical manifestation of beta-thalassemia trait cases	Number of cases (%)
Fever for evaluation	34 (46%)
Antenatal screening	19 (26%)
Generalized weakness	5 (7%)
General checkup	5 (7%)
Familial screening	4 (5%)
Other diseases included tonsillitis, appendicitis, ureteric colic, and intervertebral disc prolapse	7 (9%)

The mean and SD of each hematological parameter were calculated. The mean RBC count was 5.5 million/cu.mm, MCV was 63.8 fL, MCH was 19.6 pg, RDW-CV was 17.8% and HbA2 was 5.5%. Among the 74 cases studied, 37(46%) cases had Hb more than >11 g/dL, 22 cases had mild anemia, 12 cases had moderate anemia and three cases had severe anemia as shown in Table 3.

**Table 3:** Characterisation of RBC parameters in beta-thalassemia trait cases Hb: hemoglobin; SD: standard deviation; RBC: red blood cell; MCV: mean corpuscular volume; MCHC: mean corpuscular hemoglobin concentration; RDW: red cell distribution width; HbF: fetal hemoglobin; dL: decilitre; fL: femtolitres; pg: picogram; CV: coefficient of variation

Parameters	Hb (g/dL)	RBC million/cu.mm	MCV (fL)	MCH (pg)	MCHC (%)	RDW CV (%)	HbA2 (%)	HbF (%)
Minimum	4.8	1.6	52.4	14.6	25.6	13.5	4.1	<0.8
Maximum	17.1	7.6	102.4	31.1	34	29.5	7.1	5.9
Mean	10.8	5.5	63.8	19.6	30.6	18.0	5.5	1.9
SD	2.2	1.2	7.5	2.5	1.5	2.7	0.6	1.2
Normal reference range	Male (13-17) and female (12-15)	Male (4.5-5.5) and female (3.8-4.8)	83-101	27-32	31.5-34.5	11.6-14	1.5-3.5	0-2

Out of the 74 cases studied, 34 cases had Hb levels higher than 11 g/dL, 22 cases had mild anemia, 12 cases had moderate anemia, and three cases had severe anemia. There were no cases of very severe anemia in our study. The clinical manifestations of these patients are shown in Table 4. 

**Table 4:** WHO grading of anemia in beta-thalassemia cases with its clinical manifestations WHO: World Health Organization

WHO grading of anemia	Hb (g/dL)	Beta-thalassemia trait cases (n)	Clinical details
Very severe	<4	Nil	Nil
Severe	<7	3	Antenatal-2 cases, generalized weakness and splenomegaly-1 case
Moderate	7-9	12	Antenatal-11 cases, generalized weakness-1 case
Mild	9-11	22	Antenatal-6 cases, fever-6 cases, general checkup-5 cases, familial screening-3 cases, tonsillitis-1 case, appendicitis-1 case

Table 5 provides a summary of the mean, minimum value, maximum value, and SD for WBC and platelet count. Out of 74 cases, three patients with platelet count was less than 50,000 cells/cu.mm. In 58 cases, the platelet count was more than 1.5 lakhs/cu.mm.

**Table 5:** Characterisation of WBC and platelet parameters in beta-thalassemia trait cases WBC: white blood cell; SD: standard deviation

Parameter	WBC (cells/cu.mm)	Neutrophils (%)	Lymphocytes (%)	Monocyte (%)	Eosinophil (%)	Basophil (%)	Platelet count (lakhs/cu.mm)
Minimum	2840	27.5	6.6	1.0	0	0	0.06
Maximum	19300	92	57.3	9.9	19.6	1.1	4.1
Mean	9170	64.9	25.7	4.7	3.5	0.3	2.4
SD	3099	12.7	10	1.9	3.7	0.2	1.0
Normal reference range	4000-1000	40-80	20-40	2-10	1-6	<1-2	1.5-4.5

Table 6 depicts the distribution of the beta-thalassemia trait in the various regions of Tamilnadu as well as in the population of other states that migrated to Tamilnadu. A 43 cases of the beta-thalassemia trait were found in Tamilnadu, among which the highest number of cases was recorded in Thiruvallur and Kancheepuram. 

**Table 6:** Distribution of beta-thalassemia trait in various places of Tamilnadu and population of other states

Tamilnadu	Number of beta-thalassemia trait cases	Migrant population other than Tamilnadu	Number of beta-thalassemia trait cases
Thiruvallur	14	Orissa	10
Kancheepuram	14	Bihar	5
Chennai	8	Jharkhand	4
Vellore	3	Tripura	3
Thiruvannamalai	2	West Bengal	3
Dindigul	1	Assam	2
Erode	1	Andhra	2
		Kerala	1
		Bangladesh	1
Total number of cases	43	Total number of cases	31

## Discussion

The beta-thalassemia was the most commonly inherited hematological quantitative disorder defined by reduced or absent globin production. Four globin chains, two alpha (α) and two beta (β) subunits each, together constitute adult HbA. Alpha-/beta-globin chains become unbalanced due to a deficiency in beta-globin quantity, which hampers the synthesis of functional HbA [13]. Defects in one of the HBBs were linked to the beta-thalassemia phenotype. The Indian subcontinent, Asia, the Middle East, and tropical Africa were all hotspots for thalassemia syndrome. In India, the prevalence of beta-thalassemia was estimated to be 3%-4% overall, and approximately 10,000-12,000 babies were born with beta-thalassemia major annually. Beta-thalassemia trait occurrences varied from under 1% to 17%, with an average of 3.3%. The Thalassemia International Federation (TIF) published the 2021 guidelines for the management of transfusion-dependent thalassemia (TDT), which categorizes beta-thalassemia into two primary groups based on the severity of the disease's clinical manifestation and the need for transfusions. There was an increased risk of beta-thalassemia major in communities where consanguineous marriage was common. This emphasizes how crucial genetic screening and counseling were for these kinds of people. Certain countries such as Cyprus, Saudi Arabia, Iran, and others have implemented premarital and prenatal screening for beta-thalassemia traits mandatory to lower the frequency. All the suspected cases that were refractory to anemia treatment and cases with the Mentzer index <13 and extended family screening are to be evaluated for HPLC screening. 

The prevalence of beta-thalassemia trait varied from place to place due to migration. Our study was carried out in the outskirts of Chennai, where there are more industries and a higher influx of migrant workers. The occurrence of beta-thalassemia trait in our study showed 74 cases (8.8%). Our findings were consistent with a study carried out in Chennai by Bhuvana et al., which revealed that out of 5207 cases screened, 387 cases (7.4%) tested positive for the beta-thalassemia trait and also, they identified 30 different mutations by doing a molecular analysis study [14]. In the study conducted by Saxena et al. Gujarat showed that among 1236 cases of all ages screened, 495 cases (40.01%) were positive for the beta-thalassemia trait. Beta-thalassemia carrier diagnostic criteria included anemia with MCV <80 fL, MCH <27 pg, and HbA2 Fraction >3.5% [15]. A study carried out by Wickramaratne KAC et al. showed that among 111 cases screened, 89 cases were positive for beta-thalassemia trait [16]. The beta-thalassemia trait in Wickramaratne KAC et al. was diagnosed mainly based on the HPLC screening with HbA2 levels between 3.5% and 7.5% were considered as beta-thalassemia trait and non-beta-thalassemia trait with HbA2 levels less than 3.5%. A cross-sectional study conducted by Bhargava et al. showed the occurrence of beta-thalassemia trait cases was 6.7% as depicted in Table 7 [17].

**Table 7:** Comparison of occurrence of beta-thalassemia trait in various studies

Distribution of cases	Present study	Bhuvana Selvaraj et al. study in Chennai (2016) [14]	Saxena s et al. study in Gujarat (2020) [15]	Wickramaratne KAC et al. study (2021) [16]	Bhargava M et al., study (2020) [17]
Number of beta-thalassemia trait	74 (8.8)%	387 (7.4)%	495 (40.1%)	89	6.7 (%)
Total number of cases screened	837	5207	1236	111	1353
Age group involved in years	18-56	-	-	Jan-74	>18
Mean age in years	27	-	-	-	-
Males (number of cases)	42	157	-	26	30
Females (number of cases)	32	230	-	63	61

This aligns with Wickramaratne KAC's observation that not all cases of beta-thalassemia trait exhibit lower Hb levels. In terms of hematological parameters, our study demonstrated similar findings to Wickramaratne KAC et al., with mean Hb at 10.8g/dL, RBC count at 5.5 million/cu.mm, MCV at 63.8 fL, and MCH at 19.6 pg. Bhargava et al.'s study reported mean Hb at 12g/dL and RBC count at 5.1 million/cu.mm while Jameel et al.'s findings showed a mean RBC count of 4.1 million/cu.mm and a mean RBC count was 4.1 million/cu.mm and Elshaikh RH et al. study in 2022 demonstrated a mean RBC count of 5.7 million/cu.mm and RDW 18.4% [18,19]. In comparing our findings with previous studies, it was evident that there were variations in hematological parameters among individuals with beta-thalassemia trait. While some studies, such as Bhargava et al., reported higher mean Hb levels and MCH values, others like Jameel et al. demonstrated lower Hb levels and MCV values. RDW % in our study was in concordance with Elshaikh RH et al. study (Table 8).

**Table 8:** Comparison of RBC parameters in various studies Hb: hemoglobin; RBC: red blood cell; MCV: mean corpuscular volume; MCH: mean corpuscular hemoglobin; RDW: red cell distribution width; SD: standard deviation; dL: decilitre; fL: femtolitre; pg: picogram

Studies	Hb (g/dL) mean±SD	RBC (million/cu.mm) mean±SD	MCV (fL) mean±SD	MCH (pg) mean±SD	RDW (%) mean±SD
Present study	10.8±2.2	5.5±1.2	63.8±7.5	19.6±2.5	18.0±2.7
Wickramaratne KAC et al. study (2021) [16]	10.45±1.6	5.3±0.8	62.1±5.4	19.7±1.7	15.9±2.3
Bhargava M et al. study (2020) [17]	12±0.4	5.1±0.6	65±4.6	24.2±3.4	15.9±2.3
Jameel et al. study (2017) [18]	7.2±0.7	4.1±0.6	73.5±1.3	18.8±1.8	16.5±1.8
Elshaikh RH et al. study (2022) [19]	10.9±1.8	5.7±0.8	59.1±6.5	19.1±2.9	18.4±2.8

In this study, the maximum number of beta-thalassemia trait cases (54%) showed HbA2 levels between 5.1 and 6 as shown in Table 9, which was in concordance with Bhuvana et al. [14].

**Table 9:** Distribution of HbA2 fractions among beta-thalassemia trait cases HbA2: hemoglobin A2

HbA2	Present study	Bhuvana et al. study Chennai (2022) [14]
4-4.5	2 (2.7%)	40 (10.34%)
4.51-5.0	13 (17.6%)	107 (27.65%)
5.1-6.0	40 (54)	194 (50.3%)
>6.1	19 (25.7)	46 (11.89%)
Total	74	387

The majority of the individuals with beta-thalassemia trait explored from Tamilnadu were specifically from the districts of Thiruvallur and Kanchipuram, followed by Chennai. Cases other than Tamilnadu belonged to the migratory population; the majority were from Odisha and Bihar. These differences may stem from various factors including demographics, genetic variations, and environmental influences. Understanding these variations is crucial for accurate diagnosis and management of beta-thalassemia trait, highlighting the importance of further research in this field to enhance our comprehension and clinical practices.

Limitation

Our study was conducted in a single center and the sample volume was small, which suggests a pathway for future research, including the need for multicentric studies to validate and extend the findings.

## Conclusions

Thalassemia can indeed pose challenges not only for affected families but also for society. The study also concluded that regular monitoring of RBC indices, RBC count, and Mentzer Index along with HPLC analysis is an effective method to diagnose hidden beta-thalassemia trait cases. Increasing awareness among hematopathologists and general practitioners about RBC indices and HPLC can significantly enhance the detection of beta-thalassemia trait cases. We observed that the majority of patients arriving at the hospital presented with fever and were seeking evaluation, with many of them lacking knowledge about beta-thalassemia trait, followed by antenatal cases who came for routine check-ups. Our study also suggests regular screening of antenatal mothers and creating awareness about beta-thalassemia among them. The screening test allows for appropriate genetic counseling and informal decision-making for prospective parents, contributing significantly to the management and prevention of beta-thalassemia in newborns.

## References

[1] Jaing TH, Chang TY, Chen SH, Lin CW, Wen YC, Chiu CC (2021). Molecular genetics of β-thalassemia: a narrative review. Medicine (Baltimore).

[2] Rao E, Chandraker SK, Singh MM, Kumar R (2024). Global distribution of β-thalassemia mutations: an update. Gene.

[3] Sanchez-Villalobos M, Blanquer M, Moraleda JM, Salido EJ, Perez-Oliva AB (2022). New insights into pathophysiology of β-thalassemia. Front Med (Lausanne).

[4] Lee JS, Rhee TM, Jeon K (2022). Epidemiologic trends of thalassemia, 2006-2018: a nationwide population-based study. J Clin Med.

[5] Farmakis D, Porter J, Taher A, Domenica Cappellini M, Angastiniotis M, Eleftheriou A (2022). 2021 thalassaemia International Federation guidelines for the management of transfusion-dependent thalassemia. Hemasphere.

[6] Kattamis A, Forni GL, Aydinok Y, Viprakasit V (2020). Changing patterns in the epidemiology of β-thalassemia. Eur J Haematol.

[7] Basu S, Rahaman MZ, Dolai TK, Shukla PC, Chakravorty N (2023). Understanding the intricacies of iron overload associated with β-thalassemia: a comprehensive review. Thalass Rep.

[8] Yadav SS, Panchal P, Menon KC (2022). Prevalence and management of β-thalassemia in India. Hemoglobin.

[9] Ejaz S, Abdullah I, Usman M (2023). Mutational analysis of hemoglobin genes and functional characterization of detected variants, through in-silico analysis, in Pakistani beta-thalassemia major patients. Sci Rep.

[10] Kim S, AlDhaheri H, Kim Kim (2022). Going back to fundamentals: three marriageable actions for thalassemia and carrier population management. Thalass Rep.

[11] Sonkawade ND, Kinikar AA, Kulkarni RK, Dawre RM, Valvi CT, Kamath PA (2022). Screening of extended family members of thalassemia major children as a thalassemia preventive strategy. Ethiop J Health Sci.

[12] Munkongdee T, Chen P, Winichagoon P, Fucharoen S, Paiboonsukwong K (2020). Update in laboratory diagnosis of thalassemia. Front Mol Biosci.

[13] Chauhan W, Zennadi R (2023). Keap1-Nrf2 heterodimer: a therapeutic target to ameliorate sickle cell disease. Antioxidants (Basel).

[14] Selvaraj B, Subramanian G, Ramanathan SK, Soundararajan S, Narayanasamy S (2022). Studies on molecular spectrum of beta thalassemia among residents of Chennai. AIMS Mol Sci.

[15] Saxena S, Jain Jain, R R (2020). Evaluation of the diagnostic reliability of Mentzer index for beta thalassemia trait followed by HPLC. Trop J Pathol Microbiol.

[16] Wickramaratne KAC, Wijewickrama Wijewickrama, DC DC (2021). Screening for beta-thalassemia trait; applicability of red cell indices and parameters - a study in Sri Lanka. Int J Health Sci (Qassim).

[17] Bhargava M, Kumar V, Pandey H, Singh V, Misra V, Gupta P (2020). Role of hematological indices as a screening tool of beta thalassemia trait in eastern Uttar Pradesh: an institutional study. Indian J Hematol Blood Transfus.

[18] Jameel T, Baig M, Ahmed I, Hussain MB, Alkhamaly MB (2017). Differentiation of beta thalassemia trait from iron deficiency anemia by hematological indices. Pak J Med Sci.

[19] El Sheikh RH, Amir R, Ahmeide A (2022). Evaluation of the discrimination between beta-thalassemia trait and iron deficiency anaemia using different indexes. Int J Biomed.

